# Supplementary material for: Ovarian Endometrioid and Clear Cell Carcinomas with Low Prevalence of Microsatellite Instability: A Unique Subset of Ovarian Carcinomas Could Benefit from Combination Therapy with Immune Checkpoint Inhibitors and Other Anticancer Agents
Source: Healthcare (Basel). 2022 Apr 7;10(4):694. doi: 10.3390/healthcare10040694 (PMC9032309; doi:10.3390/healthcare10040694)
Supplement: Supplementary file 1 [file healthcare-10-00694-s001.zip › healthcare-1603752 supplementary.pdf]

healthcare

MDPI

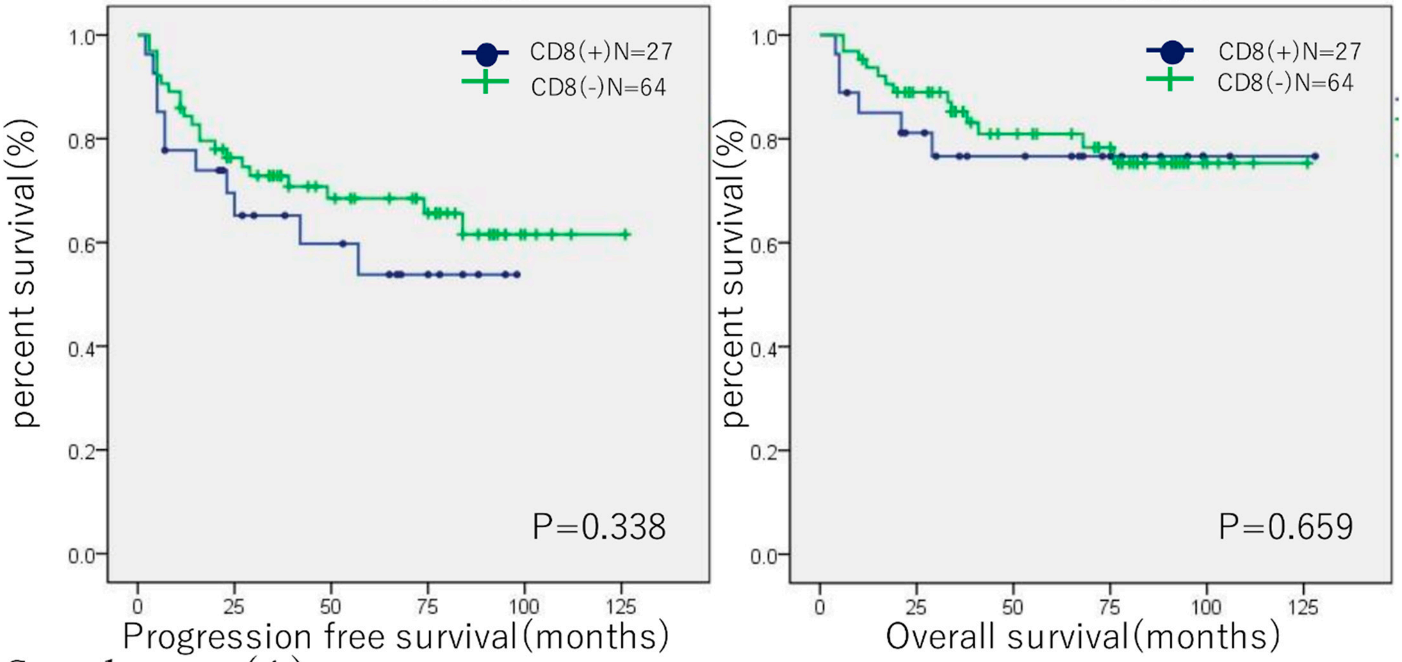

Supplyment(A)

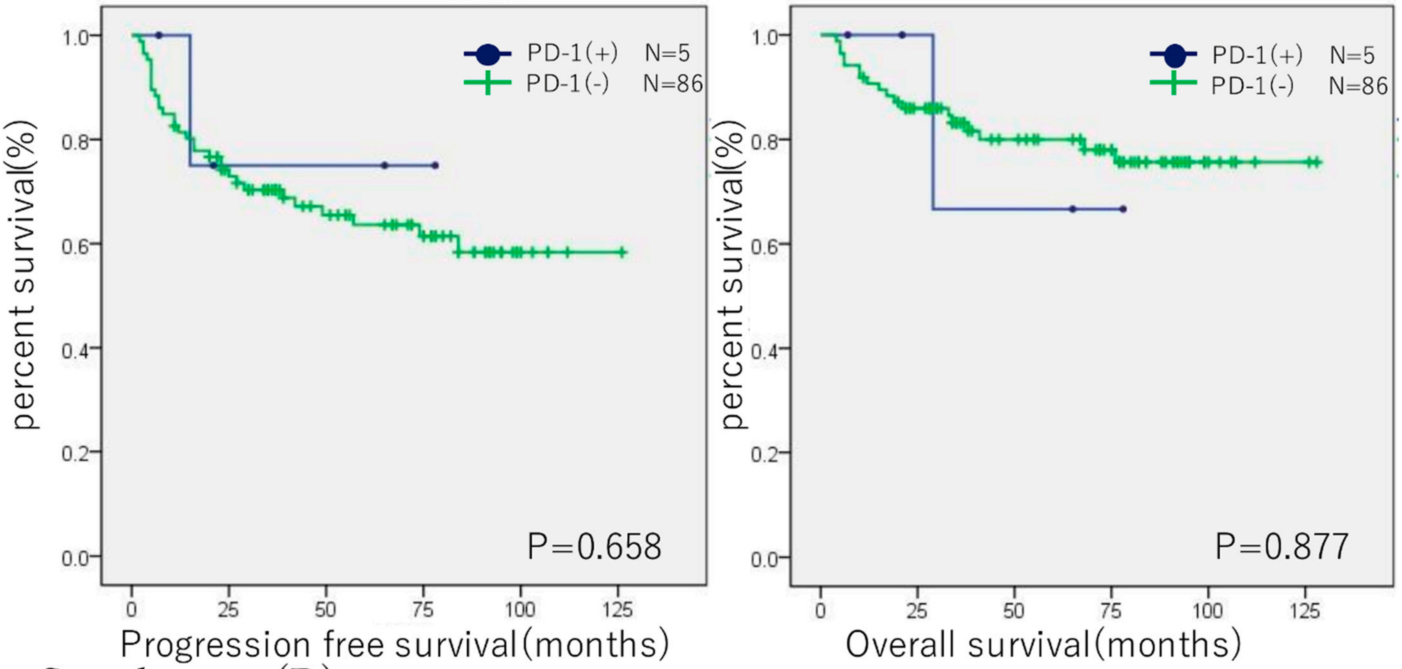

Supplyment(B)

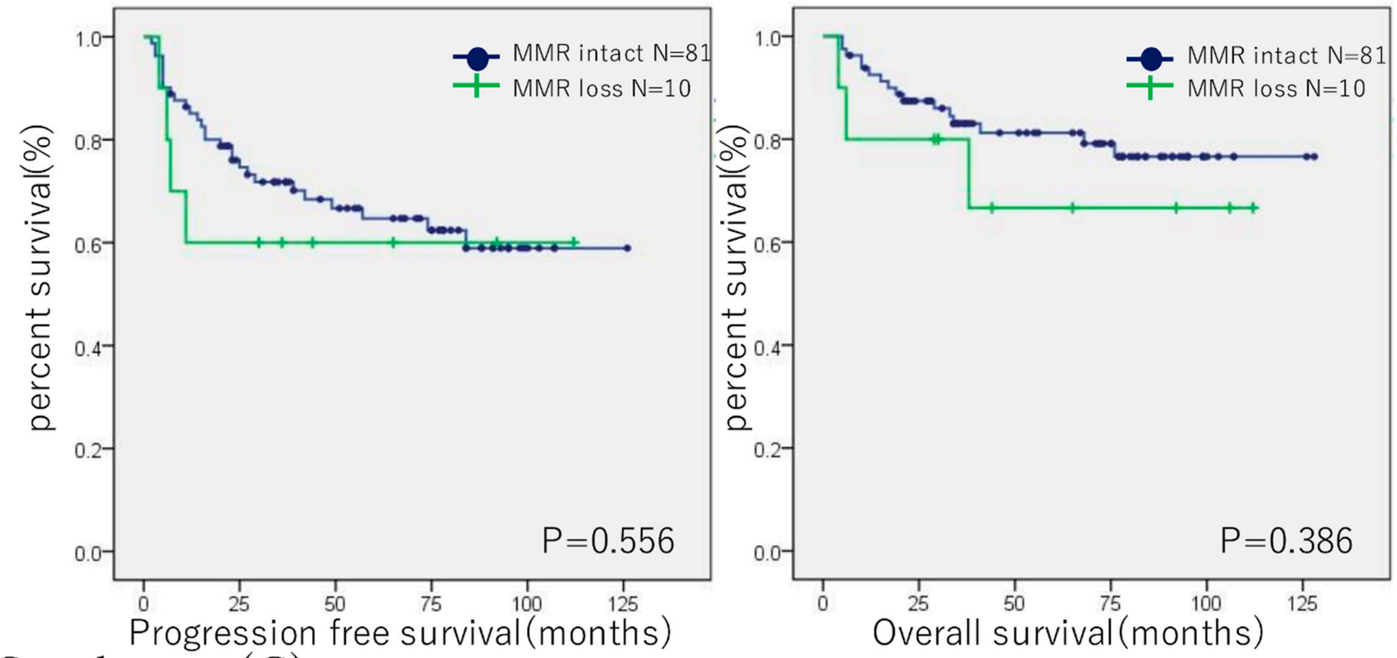

Supplyment(C)

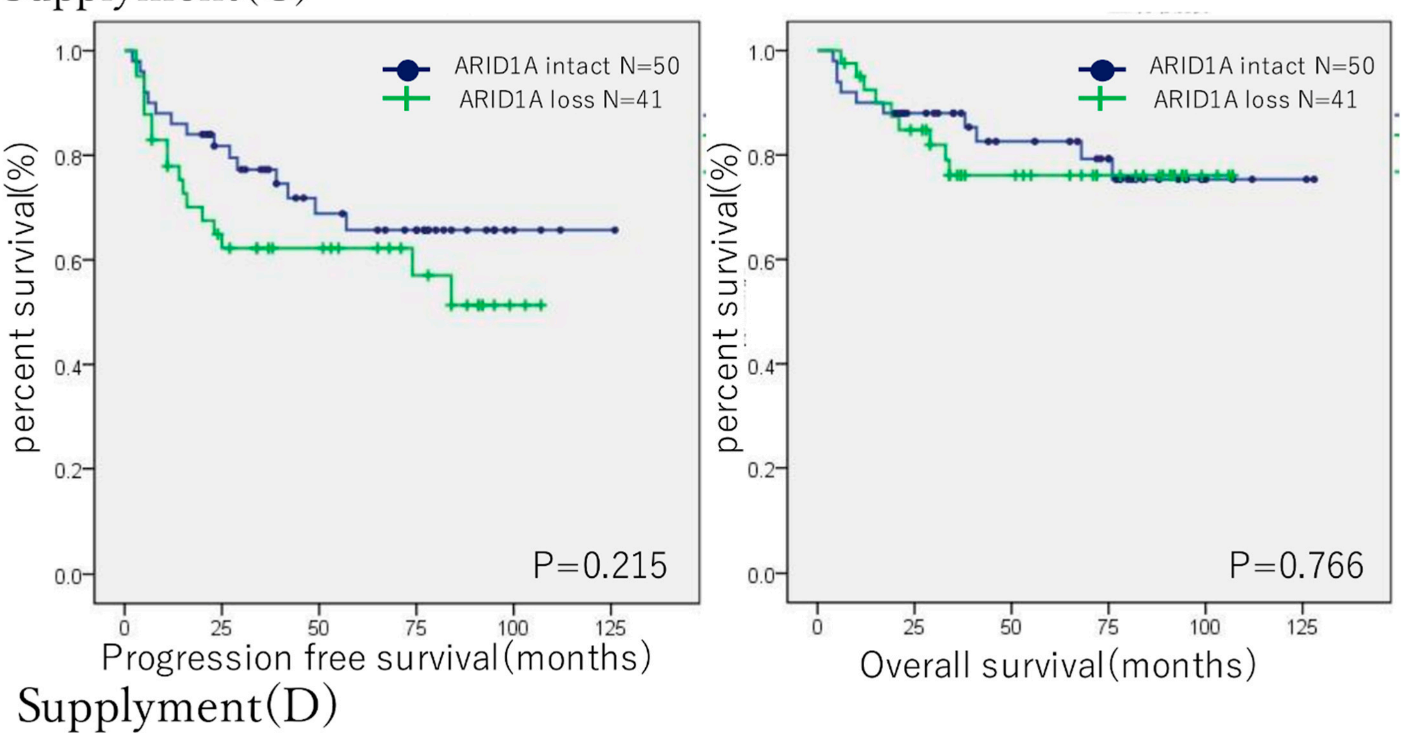

Supplyment(D)

Figure S1. (**A**) Progression-free (left panel) and overall (right panel) prognostic analysis on CD8 expression status in ovarian endometrioid and clear cell carcinomas. (**B**) Progression-free (left panel) and overall (right panel) prognostic analysis on PD-1 expression status in ovarian endometrioid and clear cell carcinomas. (**C**) Progression-free (left panel) and overall (right panel) prognostic analysis on MMR protein expression status in ovarian endometrioid and clear cell carcinomas. (**D**) Progression-free (left panel) and overall (right panel) prognostic analysis on ARID1A expression status in ovarian endometrioid and clear cell carcinomas.
